# Harnessing Single-Cell and Spatial Transcriptomics for Crop Improvement

**DOI:** 10.3390/plants13243476

**Published:** 2024-12-11

**Authors:** Yuzhao Hu, Linkan Dash, Gregory May, Nagesh Sardesai, Stéphane Deschamps

**Affiliations:** Corteva Agriscience, Johnston, IA 50131, USA; yuzhao.hu@corteva.com (Y.H.); linkan.dash@corteva.com (L.D.); gregory.may@corteva.com (G.M.); nagesh.sardesai@corteva.com (N.S.)

**Keywords:** single-cell transcriptomics, spatial transcriptomics, crop improvement, crop transformation, abiotic stress, disease resistance, yield

## Abstract

Single-cell and spatial transcriptomics technologies have significantly advanced our understanding of the molecular mechanisms underlying crop biology. This review presents an update on the application of these technologies in crop improvement. The heterogeneity of different cell populations within a tissue plays a crucial role in the coordinated response of an organism to its environment. Single-cell transcriptomics enables the dissection of this heterogeneity, offering insights into the cell-specific transcriptomic responses of plants to various environmental stimuli. Spatial transcriptomics technologies complement single-cell approaches by preserving the spatial context of gene expression profiles, allowing for the in situ localization of transcripts. Together, single-cell and spatial transcriptomics facilitate the discovery of novel genes and gene regulatory networks that can be targeted for genetic manipulation and breeding strategies aimed at enhancing crop yield, quality, and resilience. This review highlights significant findings from recent studies, discusses the expanding roles of these technologies, and explores future opportunities for their application in crop improvement.

## 1. Introduction

Individual cells in a complex multicellular organism interact with their immediate environment. In plants, cells tend to be remarkably adaptable in their development and response to specific stimuli. Their fate varies based on their cell lineage but is also directly impacted by their location within a tissue of interest. For example, plant inflorescence architecture is influenced by the positional information of stem cell populations known as meristem and their response to specific microenvironments [1,2]. Similarly, plant immunity response may vary based on the spatial localization of cells expressing immune receptors in response to a pathogen infection [3,4]. The characterization of a plant transcriptome provides important clues on genes that are induced under specific environments and conditions. However, assessing transcriptomic responses at the whole tissue level only provides a partial account of the subtle localized variations occurring between individual cells. The emergence of single-cell transcriptomics techniques has given researchers the opportunity to capture some of those subtle variations and to fine-tune the transcriptional networks involved in cells’ response to their specific environment. Yet, this approach often ignores other factors influencing cell fate and cell response, including cell-to-cell communication, localization within tissue, and interaction with external inducers. The presence of master regulators and gene regulatory networks in multiple cell types can also confound localized signals and lead to the involuntary confluence of specific molecular mechanisms at the single-cell level [5,6]. Therefore, in addition to its transcriptional response, the localization of a cell within a tissue of interest becomes another critical component to better understand both the local and global molecular mechanisms involved in a cell’s response to specific inducers within specific tissue contexts and environments. Recently developed spatial transcriptomics techniques can address such concerns and limitations by associating transcriptional responses at the single-cell level to their respective spatiotemporal context [7]. In addition, by capturing transcript information in situ and thus bypassing the need to recover individual cells for manipulation, spatial techniques enable the characterization of gene activity at the single-cell level within their native cellular context.

Crop improvement is the process of altering existing crops by introducing new traits to improve, for example, yield, drought resistance, or disease resistance [8,9]. New traits include “input” traits, involved in protecting crop yield by facilitating its response to external abiotic and biotic factors, and “output” traits, focused on improving crop values to consumers by delivering, for example, better seed nutritional values. Crop improvement started with the selection and breeding of beneficial traits via crossing and genetic inheritance. More recently, the advent of genetic engineering tools, such as the integration of exogenous genes (or “transgene”) via transformation [10], or, more recently, the modification of endogenous genes via gene editing [11], has enabled the rapid and targeted integration of beneficial traits into crops of interest while limiting the transmission of undesired traits and yield drags often associated with classic breeding practices. The advent of next-generation DNA sequencing technologies has allowed, in recent years, the mapping of numerous quantitative trait loci (QTL) and the subsequent characterization of candidate genes via fine mapping [12,13]. However, the discovery of candidate genes via genetic mapping can take several generations and many years to accomplish, and those genes often have limited or partial impacts on complex traits. Additionally, their ectopic expression may lead to undesired phytotoxic effects, further reducing their impact and commercial viability.

The adoption of single-cell and spatial technologies promises to accelerate those practices by enabling the discovery of candidate genes (and regulatory elements) whose activity is targeted to specific cell types within a tissue or specific stages in the development of the plant. This review is aimed at providing an update on progress, both on the technologies and on their applications for gene discovery targeted towards crop improvement. The expanding roles of those technologies and future opportunities for crop improvement will also be briefly discussed.

## 2. Single-Cell and Spatial Transcriptomics Technologies in Plant Research

The study of gene expression in a cell or a tissue of interest remains one of the most fundamental aspects of characterizing an organism’s response to specific stimuli, e.g., heat, drought, or the expression of an exogenous gene of interest. Contrary to “bulk” transcriptome analysis, single-cell transcriptomics offers a precise understanding of the various transcriptional responses that may occur within individual cells or individual cell types in response to such stimuli, enabling a comprehensive analysis of the dynamics and interactions between genes within a cell [14,15]. In plants, the presence of a cell wall and difficulties in generating stable cell suspensions preclude most single-cell technologies from being used directly without prior cell handling and modification of the techniques to single protoplast sequencing [16,17] or single nuclei sequencing [18,19,20,21]. The generation of protoplasts requires enzymatic digestion of the cell wall under conditions appropriate for releasing stable protoplasts [22]. Protoplasts are widely used in plant studies to capture single-cell transcriptomes. However, methods to generate protoplasts differ among plant species and tissue types [23] and generally introduce stresses in response to treatments that will bias gene expression towards genes that need to be subsequently removed from post-sequencing analysis [24,25]. As a result, single-nuclei RNA sequencing (RNA-seq) has become a viable alternative for single-cell transcriptomics analysis, with the obvious limitation that only nuclear transcripts (and precursors) are captured, thus providing only a partial snapshot of the overall dynamics of expression occurring within the whole cell [26].

**Figure 1:**
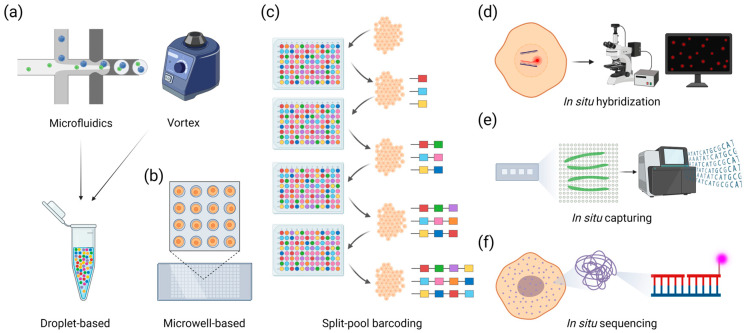
Single-cell and spatial transcriptomics technologies. (**a**–**c**) Single-cell transcriptomics technologies use specific barcodes to track individual cells without providing spatial localization information. (**a**) Droplet-based methods generate droplets containing individual cells or nuclei and the enzymatic machinery required for their processing. (**b**) Microwell-based methods isolate individual cells into separate microwells. (**c**) Split-pool barcoding methods involve multiple rounds of splitting and pooling cells, adding barcodes at each round. (**d**–**f**) Spatial transcriptomics technologies measure gene expression while preserving the spatial context of the transcripts. (**d**) In situ hybridization methods use fluorescent DNA probes to hybridize RNA molecules and capture their locations via microscopy imaging. (**e**) In situ capturing methods permeabilize RNA from tissue sections onto capture areas with spatial barcodes, generate DNA libraries, and perform next-generation sequencing. (**f**) In situ sequencing methods hybridize probes to target RNA molecules, reverse transcribe and amplify DNA, and perform sequencing within the tissue using fluorescence as readouts. Created in BioRender. Deschamps, S. (2024) BioRender.com/d79d686 (accessed on 9 October 2024).

Several techniques have been developed for single-cell or single-nuclei sequencing. The most common one uses microfluidic devices to mix within aqueous droplets individual cells (Figure 1a), protoplasts, or nuclei with the enzymatic machinery required for cell lysis and with beads containing on their surface barcoded oligonucleotides ending with a poly d(T) sequence [27]. After lysis, poly(A) RNAs are captured and reverse transcribed into barcoded cDNA molecules that are subsequently processed for sequencing and assignment to specific genes within individual cells. Commercial versions of this method include the Chromium platform from 10x Genomics (Pleasanton, CA, USA) or the “PIPseq” method from Fluent Biosciences (Watertown, MA, USA) (recently acquired by Illumina). Because cells or nuclei are encapsulated into individual droplets, this method is highly sensitive to the presence of ambient RNA, resulting from cell death or damages incurred during tissue preparation, single-cell isolation, or nuclei preparation [28]. Ambient RNA gets randomly inserted into droplets, thus introducing noise and confounding low-level biological signals of interest. This problem is particularly acute when working with fresh frozen nuclei, as cytoplasmic and organellar RNA tend to be isolated with individual nuclei following tissue thawing and cell lysis. The risk of ambient RNA is further compounded by RNA leaking through nuclear pores or in response to damage occurring to the nuclear membrane during nuclei preparation. In some instances, droplets may also contain “doublets” (e.g., more than one cell or nuclei) whose resulting transcriptional response can be difficult to separate from a true single-cell response. Alternative cell- or nuclei-loading methods are available. For example, the “SCOPE-chip” from Singleron Biotechnologies (Köln, Germany) uses a microfluidics chip to load cells into individual microwells, claiming low doublet rates because of this distinct loading method (Figure 1b). Another method developed by Parse Biosciences (Seattle, WA, USA) uses a split-pool combinatorial barcoding approach to label individual transcripts, using individual cells instead of an extraneous carrier, such as droplets or microwells, for reverse transcribing RNA into individually barcoded cDNAs (Figure 1c). The process is followed by a series of barcode ligation reactions after pooling and splitting cells or nuclei, thus creating sequence signatures that are unique to transcripts originating from individual cells or nuclei.

Single-cell or single-nuclei RNA-Seq data are often interpreted through dimension reduction algorithms such as Uniform Manifold Approximation and Projection (UMAP), which transform sparse and complex multigenic data into interpretable visualizations for genes exhibiting variable cell-specific expression [29]. Cells exhibiting similar patterns of expression are grouped together into clusters whose identity can then be further associated with specific cell types or behaviors. Clustering generally is guided by major factors of differentiation within a group of cells (including protoplasting) and, therefore, often ignores more subtle differences in gene expression that may be driven by factors such as protein diffusion or cell-to-cell communication and, more generally speaking, by local contexts surrounding cells of interest. Capturing such information requires a better understanding of the spatial organization of cells within a tissue. The combination of gene expression analysis with the spatial localization of transcripts can be captured through methods grouped under the “spatial transcriptomics” moniker. Extensive descriptions of the technological landscape and applications have been written elsewhere [7,30,31,32]. Therefore, only a brief description will be provided here, along with examples of platforms and protocols used in plants, to further illustrate the data and results being described in the present review.

The first technique, in situ hybridization, requires the hybridization of gene-specific probes to transcripts of interest, followed by the serial hybridization of secondary dye-labeled “readout” probes (Figure 1d). The tissue section is then imaged under a fluorescence microscope to localize transcripts in situ, thus offering a very high subcellular resolution of gene expression. This approach was first explored via methods, including single-molecule Fluorescence In Situ Hybridization (“smFISH”), targeting a small number of genes [33]. More recent methods have increased the number of targeted genes. In one, called “PHYTOmap” [34], a series of genes can be imaged via several rounds of smFISH in 3D. Instruments such as the MERSCOPE^TM^ from Vizgen (Cambridge, MA, USA) or the Molecular Cartography^TM^ from Resolve Biosciences (Monheim am Rhein, Germany) offer multiplexed combinatorial probing schemes where several rounds of hybridization and coloring enable transcripts derived from more than one gene to be imaged with the same probe at each round, eventually generating signatures that are unique to targeted genes of interest and enabling the in situ localization of transcripts derived from, on average, 100 to 1000 genes in parallel [35,36].

The second technique, in situ capturing, is commercially available through companies such as 10x Genomics (with its Visium line of products) or Curio Bioscience (Palo Alto, CA, USA) (Figure 1e). The Visium method for fresh frozen tissue requires the cryosectioning of a tissue block (embedded in optimal cutting temperature compound) and its immobilization onto a slide for fixation, staining, and bright field imaging [37,38,39]. For the detection of transcripts, gene-specific probes are replaced by barcoded oligonucleotides containing an oligo d(T) sequence and located at the surface of the slide. Those barcoded oligonucleotides are arranged into dense layers whose grouping is related to the identity of the barcode they contain. After imaging, tissue sections are treated to enable the diffusion of poly(A) RNA transcripts onto the slide and their hybridization to oligonucleotides through their oligo d(T) sequence. After hybridization, transcripts are reverse transcribed into barcoded cDNA, harvested, and sequenced. The barcode sequence information enables the assignment of transcripts to a specific area of the slide and, by extension, to the tissue they originate from. The Curio Bioscience method uses similar concepts for spatially capturing transcript information. However, in that case, barcoded oligonucleotides are covalently attached to tightly packed microbeads, which are immediately recovered after attaching the tissue section to the surface of the slide and permeabilization to enable the diffusion of transcripts and hybridization to the oligonucleotides. Beads then are processed to generate barcoded cDNA via reverse transcription and amplification of the original transcripts. Contrary to the Visium method, the Curio method will only recreate an in silico version of the original tissue structure and transcript localization using transcript sequences and predetermined localization of individual barcode sequences. In situ capturing methods do not require gene-specific probes and are limited mainly to capturing transcripts with a poly(A) tail. Therefore, spatial expression patterns from thousands of genes can be characterized in parallel. However, the resolution of in situ capturing methods is limited by the resolution of barcoded oligonucleotide groupings onto slides. For instance, Visium slides for fresh frozen tissue have oligonucleotide grouping within 55 μm resolution, thus limiting localization of transcripts to within distinct 55 μm regions. (Curio slides contain oligonucleotides at the surface of tightly packed 10 μm microbeads.) To address this limitation, 10x Genomics has developed a new platform called Visium HD, where dense layers of barcoded oligonucleotides are grouped within 2 μm patterns. Contrary to the Visium fresh frozen approach, this method, compatible with FFPE blocks, requires the use of whole-transcriptome pre-designed probes [40]. For now, outside of custom probe sets that aim to target a few hundred genes, this method is limited to human and mouse whole transcriptomes whose probes are directly available from 10x Genomics. Curio Bioscience also currently offers through an early access program a single-cell solution for spatial transcriptomics derived from the recently published “slide-tag” approach [41]. In there, UV-photocleavable barcoded oligonucleotides containing signature barcode sequences are first cleaved after exposure to UV, then diffuse directly into cells contained within a tissue section deposited directly on top of a slide. Nuclei retrieved from the tissue section via mechanical and enzymatic dissociation are then processed for single-nuclei RNA-seq, producing two types of sequences, one derived from poly(A) transcripts and another derived from the cleavable oligonucleotides, whose distinct barcodes enable sequences to be assigned to specific areas on the original slide. Finally, another method, spatially enhanced-resolution omics-sequencing (Stereo-Seq) from MGI [42], uses DNA “nanoballs” (DNB) to capture and localize transcripts. DNBs are first generated via rolling circle amplification of circularized oligonucleotides containing poly d(T) and barcode sequences, then docked into a grid pattern to form Stereo-seq capture chips. The resulting resolution of DNBs (0.2 μm) is significantly better than any other in situ capturing methods and technologies discussed so far.

The third technique, in situ sequencing, also requires the attachment of cryosections to slides for imaging and sequencing (Figure 1f). In that case, however, sequencing is performed in situ, following probe hybridization and extension. Briefly, in situ sequencing entails the hybridization of DNA padlock probes containing gene-specific barcode sequences directly to transcripts or to reverse transcribed cDNA [43]. Padlock probes are then ligated at the site of hybridization and used as a template for rolling circle amplification (RCA). Repeated imaging of the amplified gene-specific barcode sequence via sequencing-by-ligation (FISSEQ, STARmap) [44,45] or sequencing-by-synthesis (BaristaSeq) [46] chemistries allows for a direct readout of transcript localization within the cell. A commercial platform, Xenium^TM^ from 10x Genomics, combines gene-specific padlock probe hybridization with sequencing-by-hybridization of successive dye-labeled readout probes to determine the identity of gene-specific barcodes built into the original padlock probe. The specificity of in situ sequencing is facilitated by padlock probes, where hybridization occurs on two distinct regions of the probes. In addition, the clonal amplification of templates via RCA following circularization of the probes increases detection thresholds and, by extension, enables a high signal-to-noise ratio for imaging. However, reverse transcription of transcripts in methods using cDNA as a template, typically performed using random hexamers, tends to have poor yields of recovery. Finally, the use of gene-specific probes limits this technique to pre-determined sets of known genes in a manner similar to in situ hybridization.

In summary, all single-cell and spatial transcriptomics techniques mentioned so far rely on the use of specific or non-specific oligonucleotides hybridizing to poly(A) transcripts, enabling their characterization at the single-cell level and, in the case of spatial techniques, their localization within a tissue of interest. However, fundamental differences between those techniques suggest that each is more amenable to specific applications. While it is not in the scope of this study to highlight one platform against another, broad trends still can be highlighted. For example, single-cell and single-nuclei techniques can characterize tens or even hundreds of thousands of cells in parallel, often capturing in the process the expression pattern of hundreds or thousands of unique genes per cell. Therefore, one of the main advantages of single-cell transcriptomics lies in the very large number of cells and genes processed in parallel and, therefore, the generally high volume of information processed at once. In addition, single-cell transcriptomics is a relatively older field of study than spatial transcriptomics. Therefore, single-cell analysis can rely on an extensive portfolio of software tools and strategies honed over several years of development and practice. Single-cell sequencing also relies on the use of short-read sequencing and is, therefore, most suited for sequence-alignment-based RNA-Seq assays. However, it must be noted that recent developments in single-cell long-read sequencing, like the recently released Kinnex™ single-cell RNA kit from PacBio (Palo Alto, CA, USA), suggest that de novo characterization of isoforms is now possible at the single-cell level.

A major advantage of spatial transcriptomics over single-cell transcriptomics relies on the fact that transcripts can be localized within a tissue or a cell type of interest. The three major categories of spatial transcriptomics techniques described here (in situ hybridization, in situ capturing, and in situ sequencing) can be distinguished based on two major factors: microscopic resolution and number of transcripts characterized for a given tissue section. In the absence of known leads of interest, open systems like in situ capturing are often techniques of choice for an initial broad review of transcripts expressed in a specific region or cell type of interest. In situ capturing data, combined with single-cell analysis, can be used to discover potential leads of interest whose localization can then be further refined at the single-cell, or even subcellular, level using in situ hybridization or in situ sequencing techniques. The choice between those two often depends on more specific matters, including costs, the sensitivity of the techniques, specificity, and the ability to design probes of interest in relation to the structure of the transcripts of interest. It must be noted that those decisions often are specific to plant tissues, where whole-transcriptome probe sets for in situ sequencing or in situ hybridization are generally not commercially available, contrary to other organisms like humans or mice.

## 3. Application of Single-Cell and Spatial Transcriptomics in Crop Improvement

Single-cell and spatial transcriptomics technologies have been employed in crop species to investigate agricultural traits and provide new insights into the next generation of crop traits [47]. These technologies have been applied extensively in various aspects of crop improvement, including but not limited to crop transformation [48], disease resistance [4], abiotic stress tolerance [49], yield enhancement [50], general plant organ development [51,52], and the development of specialized crop structures [53,54].

### 3.1. Crop Transformation

Crop transformation is a crucial technology for crop genome engineering, facilitating the development of specific traits [55]. Over the past few decades, the creation and commercialization of genetically modified crops have significantly enhanced the yield stability of major crops, particularly in areas of insect control and herbicide tolerance [56]. The commercialization of these transgenic traits is closely tied to advancements in plant regeneration and crop transformation technologies [57]. The application of more recent methods for crop improvement, such as clustered regularly interspaced short palindromic repeats (CRISPR) gene editing, also heavily relies on successful crop transformation technologies to introduce gene editing tools into plant cells [58].

Somatic embryogenesis is one of the most prevalent methods of crop transformation [59]. The totipotency of somatic plant cells enables the regeneration of plant somatic cells and the development of plant somatic embryos [60]. These somatic embryos may develop from embryogenic callus tissue, typically referred to as Type II callus, and go through different stages of somatic embryogenesis, including the formation of globular proembryos and apical meristem development, followed by subsequent germination of the somatic embryos to form a shoot and root of a newly developed plant [55]. During somatic embryogenesis, it has been observed that certain developmental genes, particularly transcription factors, can significantly boost transformation efficiency [61]. However, the underlying mechanism through which these transcription factors enhance somatic embryogenesis is not fully understood [59].

It is important to note that somatic embryos often originate from a subset of cells within the transformed explant, leading to considerable heterogeneity within the transformed tissue, which consists of embryogenic and non-embryogenic cells [62]. Therefore, employing single-cell and spatial transcriptomics technologies could generate a more refined gene expression profile of the transformed tissue and provide a deeper understanding of the mechanism of somatic embryogenesis [62]. This approach could also pave the way for the discovery of novel developmental genes to enhance crop transformation. Similarly, the regeneration of shoot tissue from cultured callus tissue is a crucial step in crop transformation [63]. However, the mechanism of how the shoot organ develops during plant regeneration is not well understood [64]. The callus tissue is also heterogeneous, with some callus tissue successfully regenerating into a shoot and root to form a new plant, while other callus tissue remains in an undifferentiated stage, preventing the formation of a new transformed plant [65]. Single-cell and spatial transcriptomics technologies could enable a better understanding of the plant shoot regeneration process, which could help increase the efficiency of generating transgenic plants [66].

Single-cell RNA-seq technology has been employed to investigate the process of somatic embryogenesis, using cotton hypocotyl as the explant [62,67]. Zhu et al. examined the somatic embryogenesis process by comparing the single-cell RNA-seq profiles between two cotton genotypes: the highly regenerable line Jin668 and the recalcitrant line TEXAS MARKER-1 (TM-1) [67]. They performed a time-course single-cell RNA-seq experiment on these two genotypes, collecting cells at different time points post-callus induction. This study not only identified various cell types within the callus and their associated marker genes but also discovered two auxin-related genes, *LIKE-AUX 2* (*LAX2*) and *LAX1*, and a wound signaling-related gene, *LIPOXYGENASE 3* (*LOX3*), that are crucial for cotton hypocotyl regeneration. Furthermore, Zhu et al. experimentally validated the importance of *LAX2*, *LAX1*, and *LOX3* in cotton hypocotyl somatic embryogenesis [67]. CRISPR knockout lines of *LAX1* exhibited reduced callus growth, while overexpression of *LAX2* resulted in increased embryogenic callus production. Similarly, *LOX3* knockout led to decreased callus tissue proliferation [67].

In another study, Guo et al. performed single-cell RNA-seq on two distinct callus tissues derived from cotton hypocotyl: the embryogenic callus and the non-embryogenic callus [62]. They identified unique cell clusters corresponding to either embryogenic or non-embryogenic callus. Interestingly, they found that the expression of the *SOMATIC EMBRYOGENESIS-ASSOCIATED LIPID TRANSFER PROTEIN* (*SELTP*) gene peaked in the middle of the embryogenic pseudo-time trajectory. *SELTP* was functionally validated for its role in somatic embryogenesis, with *SELTP* silencing enhancing embryogenic differentiation and overexpression of *SELTP* leading to a loss of embryogenic competence [62]. These two studies provide valuable insights into the cellular heterogeneity of cotton hypocotyl somatic embryogenesis and identify novel genes with significant potential for optimizing the plant regeneration process and improving the efficiency of crop transformation [62,67].

The process of shoot regeneration from the callus has been studied in tomatoes using single-cell and spatial transcriptomics technologies [48]. Song et al. employed single-nucleus RNA-seq, 10x Genomics Visium, BMKMANU S1000 from BMKGENE (Beijing, China), and Stereo-seq technologies to generate a comprehensive spatial transcriptomic atlas of tomato callus shoot regeneration [48]. The high resolution of spatial transcriptomics enabled the identification of shoot primordia within the regenerating callus. It was found that the chlorenchyma cells, which are induced by light and surround the shoot primordia, play a crucial role in shoot regeneration. Interestingly, the study revealed that either dark treatment or inhibition of photosynthesis significantly impeded the shoot regeneration process in tomato callus [48]. This study underscores the functional significance of light-induced chlorenchyma cells in plant regeneration [48].

In conclusion, crop transformation technology is instrumental in crop improvement, serving not only to integrate transgenes efficiently into crop species but also to enhance gene editing efficiency [68]. The application of single-cell and spatial transcriptomics technologies facilitates a deeper understanding of the heterogeneity in gene expression among various cell types within somatic embryogenic tissue. The use of single-cell and spatial transcriptomics technologies holds significant promise for the further optimization of plant transformation processes [65].

### 3.2. Disease Resistance

Crop diseases continually pose a significant obstacle to yield stability, with a variety of diseases prevailing in different years and leading to substantial yield loss in agriculture [69]. Although fungicides can control many fungal diseases to some extent, their application can lead to environmental contamination and potential health risks to humans [70]. As a result, many countries have imposed strict regulations on fungicide use in agriculture [71]. Moreover, safe and effective chemical treatments for plant diseases caused by other pathogens, such as bacteria and viruses, are lacking [72,73]. This situation has spurred an increased demand for crops with inherent disease-resistance traits [74]. Therefore, it is crucial to deepen our understanding of disease resistance derived from plant immunity to pathogens and discover new, effective disease resistance traits in crops [75].

Plants rely on their innate immune system to fight against various pathogens [76]. Their innate immune system encompasses two pivotal components, namely pattern-triggered immunity and effector-triggered immunity [77]. Plant cells have pattern-recognition receptors that recognize specific pathogen-associated molecular patterns, thereby instigating pattern-triggered immunity [78]. However, certain pathogens have evolved sophisticated mechanisms to circumvent pattern-triggered immunity by secreting effectors into host plant cells, thereby suppressing the immune response [79]. Over time, plants have developed diverse receptors that specifically recognize these pathogen-derived effectors [80]. The activation of these effector-specific receptors, known as resistance (R) proteins, initiates another aspect of the immune system called effector-triggered immunity, providing robust resistance against invading pathogens [81].

When plants are exposed to a pathogen, the infection typically exhibits a high degree of spatial heterogeneity, with some plant cells directly interacting with the pathogen while others do not [4]. Moreover, the plant immune response is both localized and distal, including a localized hypersensitive response that induces programmed cell death in cells directly in contact with or adjacent to the pathogens, preventing the pathogen from spreading across plant tissue [82]. It also includes systemic acquired resistance that prepares distal plant cells for an enhanced immune response if the pathogen is not contained locally and spreads to other plant tissues [83]. In addition to spatial heterogeneity in plant immune response to pathogens, there is also temporal heterogeneity [84]. Plant cells initiate pattern-triggered immunity and effector-triggered immunity at different stages upon contacting pathogen cells [85]. Therefore, conventional bulk RNA-seq approaches to studying plant immune responses have many limitations, as they cannot characterize and reflect the precise heterogeneous plant immune response against various pathogens [4]. This limitation prevents the discovery of cell-type-specific novel plant genes crucial for plant resistance or susceptibility against pathogens [4]. As a result, single-cell and spatial transcriptomics technologies are highly beneficial in studying the specific and localized response in plant immune response against pathogens [86].

Several studies have utilized single-cell RNA-seq to explore plant resistance to various diseases [4]. For instance, Bai et al. used this technology to investigate the resistance of strawberries to *Botrytis cinerea*, a fungal pathogen that causes gray mold disease [87]. They collected single-cell RNA-seq data from infected strawberry leaves at different time points post-infection [87]. They annotated the cell clusters into different cell types and found that hydathode, upper epidermal, and mesophyll cells might exhibit the most significant initial response to the fungal infection [87]. Additionally, their trajectory analysis of epidermal and mesophyll cells revealed distinct changes in gene expression patterns, highlighting the spatial heterogeneity of the cellular response to the fungal infection [87]. Furthermore, they constructed a transcriptional regulatory network using their single-cell RNA-seq data [87]. They identified potential transcription factors that could be crucial for the plant disease response [87]. This study highlights the heterogeneity of the plant’s immune response across different cell types within the tissue and suggests the potential for identifying new disease-resistance genes through cell-type-specific analysis of gene expression patterns during a pathogen attack [87].

Similarly, Cao et al. utilized single-cell RNA-seq to dissect the immune response of maize roots against *Fusarium verticillioides*, the causative fungal pathogen of the severe corn disease, Fusarium stalk rot [88]. The researchers gathered single-cell RNA-seq data from two distinct maize inbred lines, Qi319 (resistant) and B104 (susceptible), at 48 h following infection [88]. Their analysis identified a cell type-specific induction of *ZmWUSCHEL HOMEOBOX 5B* (*ZmWOX5b*) and *ZmPIN-FORMED 1A* (*ZmPIN1a*) in the root apical meristem cells upon fungal challenge [88]. Functional validation confirmed that overexpressing *ZmWOX5b* or *ZmPIN1a* enhanced resistance to stalk rot, while silencing these genes aggravated the disease symptoms [88]. Furthermore, Cao et al. conducted a cell-type-specific gene co-expression network analysis, identifying *ZmPHENYL ALANINE AMMONIALYASE 6* (*ZmPAL6*), *ZmCAFFEIC ACID O-METHYLTRANSFERASE* (*ZmCOMT*), and *ZmCAFFEOYL-COA O-METHYLTRANSFERASE 2* (*ZmCCoAOMT2*) as key genes responsive to fungal infection [88]. Knockdown of these genes intensified the stalk rot phenotype relative to wild-type plants [88]. This study underscores the heterogeneity of plant immune responses to pathogens and the significant potential of single-cell RNA-seq for uncovering novel resistance genes for crop improvement [88].

In addition to fungal diseases, single-cell RNA-seq technology has been employed to investigate viral diseases in crops [89]. For instance, Yue et al. utilized single-cell RNA-seq to analyze tomato leaves, both uninfected and infected with the tomato chlorosis virus [89]. Their study revealed a dramatic change in cell composition upon virus infection, marked by a significant increase in the proportion of trichome cells post-infection [89]. Moreover, Yue et al. performed a cell cluster-specific transcription factor-target gene network analysis [89]. They discovered that the transcription factor ETHYLENE RESPONSIVE ELEMENT BINDING FACTOR 4 (ERF4) was crucial in regulating the gene expression network during viral infection [89]. They also identified *SlSKP1/ASK1-INTERACTING PROTEIN 2* (*SlSKIP2*) as a target gene of ERF4 within the gene regulatory network and demonstrated that the knockout of *SISKIP2* mitigated the leaf yellowing phenotype following viral infection [89]. This research underscores the value of cell type-specific gene transcription profiling as a powerful method for studying various crop diseases and for identifying new genes vital for plant disease resistance.

To summarize, the discovery of novel plant disease resistance traits is a pressing area of study in crop improvement. Given the high heterogeneity of plant immune responses to various diseases, both spatially and temporally, single-cell and spatial transcriptomics technologies offer a significant opportunity [4]. They can enhance our understanding of disease resistance mechanisms and aid in the identification of novel disease resistance or susceptibility genes.

### 3.3. Abiotic Stress

Climate change and global warming are urgent issues significantly impacting agricultural yields [90]. These global changes induce various abiotic stresses on crops, including drought and high-temperature stress [91]. With the continuously growing world population, the need to develop crops resilient to different abiotic stresses has increased [92]. The introduction of gene editing technology, driven by CRISPR, into crop science offers a unique opportunity to develop the next generation of crop agronomic traits, including resistance to abiotic stresses such as high temperature, drought, salt stress, and nutrient deficiencies [93]. However, our understanding of the molecular mechanisms underlying plant responses to abiotic stresses and the identification of key genes involved in abiotic stress resistance remain limited [94].

As multicellular organisms, plants rely on an intricate regulatory network comprising various cell types to coordinate responses to environmental stimuli and establish effective countermeasures to abiotic stresses [95]. Traditional bulk transcriptomic approaches to studying plant responses to abiotic stresses have the disadvantage of averaging gene expression responses across all cells within plant tissue, hindering the understanding of cell-specific responses and coordination between different cells [96]. With the advent of single-cell and spatial transcriptomics approaches, scientists can now study abiotic stress responses in crops at much higher resolution, gaining valuable insights into cell-type-specific gene expression changes and identifying potential novel genes required for plant resistance to various abiotic stresses [96]. These advancements offer great value for crop improvement in addressing climate change.

Several research groups have utilized single-cell transcriptomics technologies to investigate plant responses to abiotic stress. For instance, Chen et al. conducted a single-cell RNA-seq experiment on pea shoot apices, comparing pea plants grown under normal and boron-deficient conditions [97]. By analyzing cell type-specific gene expression changes, they observed that photosynthesis-related genes were downregulated in mesophyll cells, consistent with the reduced photosynthesis rate previously observed in boron-deficient plants [97]. Through developmental trajectory and transcription factor interaction network analysis, they discovered that the differentiation of shoot apical meristem cells into mesophyll cells involves the upregulation of chromatin remodeling genes, including homologs of the *SWITCH DEFECTIVE/SUCROSE NON-FERMENTABLE* (*SWI/SNF*) genes [97]. Importantly, they found that during boron deficiency, the expression of these chromatin remodeling genes is suppressed, leading to impaired shoot apical meristem development [97]. Utilizing single-cell RNA-seq technology, Chen et al. delineated cell type-specific gene expression changes, providing new insights into the coordinated response between different cell types in response to nutrient deficiency [97]. This study identified potential target genes for crop improvement, offering insights on how to develop plant varieties that can better thrive under nutrient-deficient conditions [97].

In another study, Li et al. used single-nucleus RNA-seq and single-nucleus assay for transposase-accessible chromatin using sequencing (ATAC-seq) to investigate the response of cotton anthers to high-temperature stress, which can cause male infertility and significant yield losses [49]. Their findings indicated that tapetal cells, essential for pollen development, were severely impacted by high temperatures [49]. By comparing single-nucleus RNA-seq data from normal and high temperatures, they observed a significant reduction in tapetal cells. More specifically, a subpopulation of the tapetal cells that are responsible for pollen wall synthesis completely disappeared under high-temperature conditions [49]. By further comparing single-nucleus ATAC-seq data from normal and high-temperature samples, they discovered that genes responsible for pollen cell wall synthesis in tapetal cells exhibited inhibited chromatin accessibility under high-temperature conditions, consistent with the single-nucleus RNA-seq findings [49]. The functional consequence of the loss of pollen cell wall synthesis gene expression in tapetal cells was evidenced by significantly thinner pollen cell walls at high temperatures [49]. Gene regulatory network analysis using the single nucleus RNA-seq data further identified *QUARTET 3* (*QRT3*) and *CYTOCHROME P450, FAMILY 703*, *SUBFAMILY A*, *POLYPEPTIDE 2* (*CYP703A2*) as key genes suppressed under high temperature, leading to abnormal pollen cell wall development and male sterility [49]. This study underscores the value of single-cell RNA-seq in elucidating cell type-specific responses to high-temperature stress and highlights novel gene targets for crop improvement [49].

Salt stress, another significant abiotic stress, has also been investigated using single-cell RNA-seq technology. Li et al. profiled the single-cell transcriptome of cotton root cells under natural growth conditions and various salt treatments [98]. By analyzing differentially expressed genes between salt-treated and control conditions, they identified cell-type-specific gene expression changes in response to salt stress [98]. Among these genes, the auxin-responsive gene *GaGRETCHEN HAGEN 3.6* (*GaGH3.6*) was functionally validated as crucial for salt stress resistance [98]. Silencing *GaGH3.6* resulted in a severe salt stress susceptibility phenotype, likely due to impaired redox processes and subsequent oxidative damage [98]. In summary, this study provided a cell-type-specific transcriptome of cotton roots in response to salt stress and identified a novel candidate gene for enhancing crop resistance to salinity stress [98].

The integration of single-cell and spatial transcriptomics technologies into the study of abiotic stress responses in crops offers significant potential for a deeper understanding of the molecular mechanisms underlying abiotic stress resistance and for identifying novel target genes for crop improvement [96]. Combining single-cell RNA-seq with single-nucleus ATAC-seq technology enhances our understanding of the molecular processes involved in abiotic stress responses, not only at the gene expression level but also at the epigenetic level [49]. With the ability to discover genes involved in abiotic stress at a cell-specific level, single-cell and spatial transcriptomics technologies are key to unlocking the next generation of gene targets for crop improvement.

### 3.4. Yield

The yield of crops is the most important agronomic trait, as it determines the economic value of many crops [99]. The yield of economically important crops, such as maize and soybean, is dependent on the phenotype of traits related to seeds or reproductive organs [100,101]. For instance, the yield of maize and soybean is measured in terms of maize kernels and soybean seeds, respectively. Therefore, studying plant seed biology is crucial for yield improvement [100,101]. In maize, the overall yield is highly influenced by the characteristics of the ear, including its length, width, and shape, which directly determine the number of kernels it can hold and, ultimately, the yield per plant [102]. A key process that affects seed yield in crops is the filling of seeds as sink tissue, mediated by the transport of photosynthetically fixed energy sources, such as sucrose, from the leaves (source) to the seeds (sink) [103]. Thus, investigating both the development of the ear primordium and the developing seed is essential for identifying novel genes that can enhance these developmental processes and improve yield [100,101].

The development of the maize ear primordium is a highly coordinated process involving three distinct types of meristems: the primary inflorescence meristem, which transitions from the shoot apical meristem; the secondary lateral spikelet pair meristem, which is produced from the inflorescence meristem; and the tertiary spikelet meristem, which is derived from the spikelet pair meristem [104]. The spikelet meristem eventually forms the floral meristems that give rise to the florets, which can potentially develop into kernels after fertilization [105]. Therefore, studying the development of the ear primordium requires delineating the different cell populations, revealing their specific spatial and temporal changes that result in the overall phenotype of the whole ear primordium [106]. Similarly, the maize kernel consists of a heterogeneous collection of different tissue and cell types, including the diploid maternal tissue called the pericarp, the triploid endosperm, and the diploid embryo [107]. The endosperm carries the most weight of the whole kernel, making it an important research subject for improving yield [108]. Given the complexity and heterogeneity of the developing maize ear primordium and kernel, the use of single-cell and spatial transcriptomics technologies offers a significant advantage for studying these tissues and holds high potential for uncovering the cell type-specific regulators of ear primordium and kernel development, guiding crop improvement in yield [50,109].

To investigate the cell-specific gene regulatory network of maize ear primordium, Xu et al. conducted a single-cell RNA-seq experiment on developing maize ears [25]. Their analysis revealed that single-cell RNA-seq data is superior to bulk RNA-seq in identifying the co-expression of functionally redundant genes that are important for maize ear development, such as *RAMOSA3* and *ZmTREHALOSE PHOSPHATE PHOSPHATASE 4* (*ZmTPP4*) [25]. This highlights the potential of single-cell RNA-seq in predicting genetic redundancy among homologous genes crucial for maize ear primordium development [25]. Additionally, Xu et al. performed a targeted genome-wide association study (GWAS) analysis by comparing marker genes from different cell clusters in the single-cell RNA-seq data with a GWAS panel of 281 maize lines for yield-related ear traits [25]. They discovered that the *ZmYABBY9* marker gene is associated with cob weight, while *RAMOSA1* and *ZmTARGET OF MONOPTEROS 5* (*ZmTMO5*) marker genes correlate with ear diameter [25]. Overall, Xu et al. characterized cell-type-specific gene expression patterns in maize ear primordium and demonstrated the potential of single-cell RNA-seq technology in identifying novel genes linked to maize yield traits [25].

Although single-cell RNA-seq reveals cell-specific gene expression patterns in the maize ear primordium, it lacks precise spatial information and relies heavily on well-characterized markers for cell type annotation [109]. Integrating single-cell RNA-seq with spatial transcriptomics can provide deeper insights into maize ear primordium development. Wang et al. combined single-cell RNA-seq with Stereo-seq spatial transcriptomics to profile cell-specific gene expression in maize ear primordium [109]. The high resolution of Stereo-seq allowed them to define expression clusters from ear primordium tissue sections and identify four new cell types in the inflorescence meristem [109]. They also distinguished gene expression units from Stereo-seq data that are associated with indeterminate and determinate meristems, identifying two MINICHROMOSOME MAINTENANCE FACTOR/AGAMOUS/DEFICIENS/SERUM RESPONSE FACTOR (MADS) box genes, *ZmMADS8* and *ZmMADS14*, as marker genes for determinate meristems [109]. Functional validation showed that *ZmMADS8* and *ZmMADS14* are essential for floral meristem development, as *Zmmads8/14* double mutants lacked normal floral meristems [109]. Additionally, Wang et al. constructed a gene regulatory network by integrating single-cell RNA-seq and Stereo-seq spatial transcriptomics data, identifying modules of co-expressed genes and their hub genes [109]. One module, enriched with trehalose-6-phosphate signal-related genes, is associated with maize yield regulation [109]. Within this module, a gene encoding a NAM, ATAF1/2, and CUC2 (NAC) transcription factor, *NACTF25*, emerged as a hub gene, highly expressed in the inflorescence meristem, suggesting its potential role in regulating trehalose-6-phosphate signal-related genes to influence maize yield [109]. Thus, by integrating single-cell and spatial transcriptomics data, Wang et al. identified novel genes crucial for maize ear primordium development and a potential regulator of yield-related traits [109].

Although the maize ear phenotype is important for yield, the maize kernel is the primary food source for direct human consumption and other agricultural uses [110]. Therefore, understanding the regulation of maize kernel development is essential for crop improvement [111]. Fu et al. conducted a spatial transcriptomics study of the maize kernel during the filling stage [50]. The spatial transcriptomics technology used in this study provided the advantage of overlapping gene expression profiles with a stained image of the kernel tissue section [50]. This enabled the researchers to assign gene expression units to specific functional compartments within the kernel [50]. One of the most important processes during the filling stage is the transportation of sucrose into the endosperm tissue for storage. Using spatial transcriptomics data, Fu et al. visually inspected the expression of *SUCROSE TRANSPORTER* (*SUT*) genes in maize and found that only *ZmSUT1* and *ZmSUT7* were specifically expressed in the basal endosperm transfer layer (BETL), which is directly in the path of sucrose transportation into the endosperm [50]. Importantly, they functionally validated the significance of *ZmSUT1* and *ZmSUT7*, as the knockdown of these genes resulted in reduced kernel weight [50]. Therefore, the application of spatial transcriptomics technology in studying the maize kernel provided valuable insights into how kernel cells coordinate spatially during the filling stage and identified novel genes that directly regulate maize yield [50].

Within the mature maize kernel, the endosperm can constitute up to 80% of the kernel’s total weight [112]. This highlights the importance of studying endosperm development to understand the overall development of the maize kernel and to enhance kernel weight for yield improvement. Yuan et al. conducted a single-cell RNA-seq study of the maize endosperm at 6 and 7 days after pollination, revealing the heterogeneity of cell types within the endosperm [113]. To construct a gene regulatory network, they also performed an optimized DNA affinity purification sequencing with polymerase chain reaction-amplified genomic DNA library (ampDAP-seq) experiment in maize endosperm, which generated extensive transcription factor-DNA binding profiles [113]. By integrating the ampDAP-seq data with the single-cell RNA-seq data, they constructed a gene regulatory network for the maize endosperm and identified specific regulons unique to each cell cluster [113]. Within these cell cluster-specific regulons, Yuan et al. identified key regulators such as the MYB-related transcription factor *MYBR29* gene, which is a crucial regulator of the BETL cell clusters [113]. They functionally validated the role of *MYBR29* in BETL development by showing that *mybr29* mutants exhibit defective BETL differentiation and a reduced kernel weight phenotype [113]. This study underscores the efficacy of high-resolution single-cell RNA-seq in identifying key regulatory genes critical for maize kernel development and yield enhancement [113].

In summary, the cellular heterogeneity within the maize ear primordium and the kernel has limited the effectiveness of conventional bulk RNA-seq approaches in identifying specific regulators of important yield-related traits. However, the advent of single-cell and spatial transcriptomics presents a significant opportunity to study the development of maize ear primordium and kernel with high resolution. By employing these advanced technologies, researchers have been able to investigate cell-specific gene expression regulation in maize ear primordium and kernel development, leading to the identification of novel genes crucial for traits related to maize yield [25,50,109,113].

### 3.5. General Plant Organ Development

Single-cell RNA-seq (scRNA-seq) and single-nucleus RNA-seq (snRNA-seq) have been instrumental in identifying key transcription factors (TFs) and signaling pathways involved in cell differentiation and development across various plant species. In the quest to understand the intricate development of peanut leaves, researchers employed high-throughput single-nucleus RNA-seq and ATAC-seq. This approach revealed a dynamic landscape of transcriptome reprogramming and chromatin accessibility across different cell types and cell cycle phases [114]. The dual-omics approach identified core TFs involved in phytohormone signaling, circadian rhythm, and plant–pathogen interactions, such as ethylene-AP2 and MYB-type TFs like *KANADI4* [114]. Additionally, *AhAT-HOOK MOTIF NUCLEAR LOCALIZED PROTEIN 11* emerged as a promoter of leaf growth and early flowering by regulating auxin pathways [114]. Further exploration using scRNA-seq of peanut leaf blades identified marker genes like *RIBULOSE-1,5-BIS-PHOSPHATE CARBOXYLASE/OXYGENASE SMALL SUBUNIT* and *LIGHT-HARVESTING CHLOROPHYLL A/B BINDING* for mesophyll cells and Phi class of *GLUTATHIONE S-TRANSFERASE* for epidermal cells, with critical TFs from the ERF and NAC families [115]. *AT-HOOK MOTIF NUCLEAR LOCALIZED PROTEIN 23* was highlighted as a positive regulator of leaf growth, promoting cell expansion and early flowering [115]. In high-oleic acid (OA) peanuts, the *FATTY ACID DESATURASE 2* gene was found to modulate cytokinin and jasmonic acid pathways, with key TFs including *WRKY6*, *ETHYLENE RESPONSE FACTOR 109*, and *MYB DOMAIN PROTEIN 102* [116]. Light and dark conditions in peanut seedlings revealed TFs like *AT-HOOK MOTIF NUCLEAR-LOCALIZED PROTEIN 17*, which regulate leaf epidermal cell enlargement [117].

Maize, a staple crop with complex developmental processes, has also benefited from the power of single-cell technologies. snRNA-seq in maize leaves identified key genes involved in stomatal movement and development, such as *ZmMUTE* and *ZmBETA CARBONIC ANHYDRASE 1* [118]. Multiplexed in situ hybridization further elucidated vein patterning genes like *PIN1a* and *TCL1/MTCP1-LIKE 1* [119]. In the ligular region, key regulators of leaf angle (LA) were identified, including *LIGULESS1*, *LIGULESS2*, and *BASIC HELIX-LOOP-HELIX 30*, with lignin deposition in adaxial sclerenchyma cells playing a critical role in restricting LA expansion [120]. In cotton, scRNA-seq and spatial transcriptomics have shed light on the mechanisms behind leaf curling. Key regulators such as *GOSSYPIUM HIRSUTUM CUP-SHAPED LEAF* and *KNOTTED1-LIKE* were identified, which modulate auxin response and cell growth to maintain auxin balance for flattened leaf blades [121]. Cassava, a vital crop for many tropical regions, has also been a subject of scRNA-seq. Researchers identified 15 unique cell populations in cassava leaves, with marker genes like *AUXIN-BINDING PROTEIN 19A* for mesophyll cells and *ALDEHYDE DEHYDROGENASE 3F1* for epidermal cells [122]. Interestingly, cassava leaves exhibit characteristics of both C3 and C4 plants, providing insights into the evolution of photosynthetic pathways [122]. In *Brassica rapa*, scRNA-seq has elucidated the differentiation between adaxial and abaxial mesophyll cells. Key regulatory genes such as *BrLIGHT-HARVESTING CHLOROPHYLL A/B BINDING 4.3* and *BrFIL.1* were identified, highlighting the functional differences between palisade mesophyll cells (PMCs) and spongy mesophyll cells (SMCs) [123].

Rice, a staple food for much of the world’s population, has also been explored using scRNA-seq. Researchers revealed widespread monoallelic gene expression in rice mesophyll cells, with key genes like *Os09g19700* [124]. In rice roots, scRNA-seq and ATAC-seq identified genes like *OsGATA BINDING PROTEIN 6* involved in cell differentiation and root architecture [125]. Single-cell transcriptomics of rice roots identified cell-type-specific genes like *LOC_Os06g38960* for the epidermis [126]. A conserved superlocus regulating root initiation was identified, with subclass IIIA genes controlling lateral roots and subclass IIIB genes regulating shoot-borne roots [127]. scRNA-seq of maize roots revealed genes like *SHORT-ROOT* and *SCARECROW* that regulate cortical complexity [128].

In rice pistils, snRNA-seq uncovered genes involved in calcium signaling and RNA processing [129]. Single-cell transcriptomics of maize shoot apices identified genes like *ZmPLASTOCHRON1* and *ZmROUGH SHEATH1*, marking the boundary between indeterminate and determinate cells and influencing leaf growth [130]. In cotton anthers, scRNA-seq revealed key TFs like *MYB DOMAIN PROTEIN 4* and *ZINC FINGER PROTEIN 1*, highlighting the impact of heat stress on gene expression and chromatin accessibility [49]. In CMS-C maize, scRNA-seq identified genes involved in mitochondrial functions and redox homeostasis, such as thiol protease and thioredoxin [131]. In maize meiosis, scRNA-seq revealed transcriptional reorganization during early prophase, with genes involved in ribosomal and mitochondrial functions [132]. In rice plumules, scRNA-seq identified genes like *OsBURP* and *OsLAZY1* involved in photosynthesis and gravitropic response [133].

The integration of single-cell and spatial omics offers a comprehensive view of the regulatory networks governing plant development, providing new insights into the roles of transcription factors, phytohormones, and other regulatory molecules. Cell-type-specific markers identified through these technologies can be leveraged to precisely target and manipulate specific cell types, thereby mitigating phytotoxicity and potential yield drag. For instance, by understanding the specific gene expression profiles and regulatory networks in different cell types, researchers can develop strategies to enhance desirable traits while minimizing adverse effects on plant health and productivity. This targeted approach can lead to the development of crops that are more resilient to environmental stresses and have improved yield potential.

Despite technical challenges such as difficulties in protoplast isolation, the need for high-resolution spatial data, and the integration of multiple omics layers, these technologies hold great promise for future research and crop improvement. As these approaches are applied to more plant species, they are expected to uncover previously unexplored mechanisms of plant development, facilitate the development of crops with improved yield and resilience, and enable the precise manipulation of key regulatory pathways for enhanced agricultural productivity.

### 3.6. Crop Specialized Structures

Advancements in single-cell and spatial transcriptomics have revolutionized our understanding of plant biology by enabling researchers to dissect complex tissues at single-cell resolution, revealing the intricate regulatory networks and cell-type-specific functions that drive growth, development, and stress responses. Here, we highlight recent studies employing single-cell and spatial transcriptomics to investigate key aspects of crop improvement in cotton, peanut, soybean, and cassava. By elucidating the molecular underpinnings of pigment gland development, fiber cell growth, fruit-pod formation, root nodule maturation, and tuberous root development, these studies provide valuable targets for genetic manipulation and breeding strategies aimed at enhancing crop yield, quality, and resilience.

To enhance pest resistance in cotton, researchers have turned their attention to the secretory glandular cells (SGCs) responsible for terpenoid biosynthesis, particularly gossypol production. A recent study employed scRNA-seq to create a detailed transcriptomic atlas of *Gossypium hirsutum* leaf cells. They identified 14 distinct cell clusters, including SGCs, and discovered that terpenoid biosynthesis is regulated by two transcription factors, *GoHEAT SHOCK TRANSCRIPTION FACTOR A4A* and *GoNAC DOMAIN-CONTAINING PROTEIN 42*, activated by *GoPIGMENT GLAND FORMATION* [134]. These findings, validated through virus-induced gene silencing (VIGS) and CRISPR–CRISPR-associated protein 9 (Cas9)-mediated genome editing, suggest that manipulating these transcription factors could increase terpenoid production, thereby enhancing the plant’s natural defense mechanisms [134]. Building on this foundation, another study delved deeper into the developmental trajectory of pigment glands. Using scRNA-seq, researchers identified two critical stages: the differentiation of young pigment gland parenchyma into central secretory cells and mature gland parenchyma, followed by the autolysis of secretory cells to release gossypol [135]. Key transcription factors such as *GoPIGMENT GLAND FORMATION*, *GbETHYLENE-RESPONSIVE TRANSCRIPTION FACTOR 114*, *GbZINC FINGER OF ARABIDOPSIS THALIANA 11*, and *GbNAC WITH TRANSMEMBRANE MOTIF 1-LIKE TRANSCRIPTION FACTOR 9* were found to regulate this process [135]. The integration of bulk and single-cell sequencing data revealed light- and gibberellic acid (GA)-mediated pathways that promote pigment gland development, providing new targets for genetic manipulation [135]. These insights pave the way for breeding cotton varieties with optimized pigment gland traits, balancing pest resistance with reduced gossypol toxicity for safer cottonseed utilization [135]. Further extending these findings, researchers compared gland cotton ‘CCRI12’ with glandless cotton ‘CCRI12gl’. They identified 12 cell clusters and pinpointed cluster 9 as pigment gland cells (PGCs) expressing *GoPIGMENT GLAND FORMATION* [136]. Pseudotime analysis suggested that PGCs differentiate from primordial cells (PRCs) [136]. The study identified 9325 differentially expressed genes (DEGs) across cell clusters, with 1430 DEGs specific to PGCs, including known regulators like *GhETHYLENE-RESPONSIVE TRANSCRIPTION FACTOR 105*, *ETHYLENE-RESPONSIVE TRANSCRIPTION FACTOR 13*, *MYB DOMAIN PROTEIN 14*, *Gh_A05G3906*, and *GhJUNGBRUNNEN1* [136]. These scRNA-seq data provide a valuable resource for developing glandless cotton mutants, facilitating the breeding of cotton varieties with reduced gossypol toxicity [136]. By targeting these specific genes, breeders can create cotton plants that are both pest-resistant and safe for human and animal consumption [136].

The intricate process of fiber cell growth in cotton has been illuminated through scRNA-seq and chromatin accessibility analyses. In one study, researchers developed a novel PTED method to prepare high-quality protoplasts from cotton ovule cells. This enabled the discovery of circadian clock-controlled rhythmic growth in early-stage fiber cells [137]. Core-clock oscillators, such as *PSEUDO-RESPONSE REGULATORs* and *CIRCADIAN CLOCK ASSOCIATED1*, along with clock-controlled genes like *GhRAPID ALKALINIZATION FACTOR 1* (*GhRALF1*) and *GhTEOSINTE BRANCHED*, *CYCLOIDEA*, and *PCF 14* (*GhTCP14*), were identified as key regulators [137]. *GhRALF1* modulates fiber growth by regulating proton pump and auxin signaling, while *GhTCP14* influences translation and mitochondrial energy [137]. This study proposed a decentralized hierarchy of circadian oscillators in fiber cells, suggesting that reprogramming these circadian networks could improve fiber yield and quality [137]. Preliminary experiments indicated that varying photoperiods affect fiber length, supporting the hypothesis that circadian clock adjustments could enhance agronomic traits [137]. By manipulating these circadian genes, it may be possible to optimize fiber growth cycles, leading to longer and stronger cotton fibers [137]. Complementing this, another scRNA-seq study focused on fiber cell initiation in cotton, addressing challenges in cell type definition and spatial transcriptome technology [54]. By analyzing fiberless mutant ovules, researchers confirmed the reliability of scRNA-seq in identifying fiber cell clusters [54]. The developmental trajectory of fiber cells was delineated, identifying key regulators at different stages: *HEADING DATE 1* and *PROTODERMAL FACTOR 2* in early stages, *MYB25-like* for differentiation, and *HOMEOBOX 3* (*HOX3*) for tip-biased diffuse growth [54]. The “relay race” model proposed that *MYB25-like* and *HOX3* act as commanders in fiber development [54]. Comparative analysis with *Arabidopsis* trichomes suggested conserved regulatory mechanisms [54]. This research provides a detailed understanding of fiber initiation, offering new targets for genetic manipulation to enhance cotton fiber quality and yield [54].

The development of peanut fruit pods has been explored through snRNA-seq and ATAC-seq, revealing the complex regulatory networks involved [53]. Researchers identified genes associated with gravitropism, such as *PIN-FORMED 1*, *NAKED PINS IN YUCCA MUTANTS 5*, *SHOOT GRAVITROPISM 6*, *INDETERMINATE DOMAIN 14*, ATP-binding cassette (ABC) transporters *ABCI11*, and *ABCB15*, specifically expressed in peg vascular cells [53]. Additionally, genes related to light and auxin responses, including *GTE1*, *PHOTOTROPIN 1*, *SUPPRESSOR OF PHYA-105*, *YABBY 5*, *AUX/INDOLE-3-ACETIC ACID 9*, *AGAMOUS-LIKE 1*, and *AGAMOUS-LIKE 5*, were differentially expressed in various cell types within the pod [53]. The study highlighted the role of MADS-box genes in spongy parenchyma cells, which are crucial for pod enlargement [53]. The integration of snRNA-seq and ATAC-seq data provided insights into the transcriptional regulation and spatial distribution of these genes, revealing complex interactions between light, gravitropism, and hormone signaling pathways [53]. This comprehensive molecular profiling offers valuable targets for genetic manipulation to improve peanut quality and yield [53]. By targeting these specific genes, breeders can enhance pod development, leading to higher yields and better-quality peanuts [53]. Expanding on these findings, another study employed Stereo-seq to analyze the spatial gene expression profiles of peanut tissues, including roots, stems, hypocotyls, and pegs [138]. Researchers identified 18 cell clusters and their spatial distribution, revealing significant cell heterogeneity [138]. For instance, the epidermis and exodermis of stems and hypocotyls were classified into different subclusters, reflecting their distinct developmental and environmental adaptations [138]. The study also identified genes related to photosynthesis, cell wall modification, and disease resistance, such as *WALLS ARE THIN 1*, *CELLULOSE SYNTHASE A7*, *XYLOGLUCAN ENDOTRANSGLUCOSYLASE/HYDROLASE 1*, *MADS-BOX PROTEIN 1*, *AGAMOUS-LIKE MADS-BOX PROTEIN 5*, *RESPONSIVE TO DEHYDRATION 22*, *BOWMAN-BIRK INHIBITOR 1*, *MAJOR LATEX PROTEIN-LIKE PROTEIN 43*, and *ABSCISIC ACID-RESPONSIVE 17* [138]. Notably, the peg’s tip and basal parts exhibited different gene expression profiles, with the tip enriched in glycoside and saponin synthesis genes, potentially protecting ovules from underground pests [138]. This high-resolution spatial transcriptome atlas provides a valuable resource for understanding cell-type-specific functions and interactions in peanut tissues, offering new insights into peanut geocarpy and potential targets for crop improvement [138]. By leveraging these insights, breeders can develop peanut varieties with enhanced resistance to environmental stresses and pests, leading to more robust and productive crops [138].

The maturation of soybean root nodules, crucial for nitrogen fixation, has been elucidated through integrated snRNA-seq and Stereo-seq [139]. Researchers identified 15 cell clusters, including root and nodule-specific cell types, and further classified these into subclusters using spatial data [139]. Uninfected cells (UCs) and infected cells (ICs) in the central infected zones (CIZ) of nodules were identified, revealing their differentiation trajectories [139]. The study highlighted the role of ureide biogenesis and transportation genes in UCs and identified subclusters involved in energy supply for symbiotic nitrogen fixation [139]. In ICs, two subtypes with distinct functions were discovered, including genes involved in infection thread formation and symbiosome membrane protein expression [139]. The study also validated the role of a subcluster-specific gene, *GLYMA_02G004800*, in nodule maturation using CRISPR-Cas9 [139]. This integrated approach provides a comprehensive cellular atlas of soybean nodules, offering valuable insights into the regulatory networks and potential targets for enhancing nitrogen fixation and crop productivity [139]. By targeting these specific genes, breeders can enhance the efficiency of nitrogen fixation, leading to more sustainable and productive soybean crops [139]. Further advancing our understanding, another study utilized snRNA-seq to construct a detailed transcriptomic atlas of soybean roots and nodules, identifying six nodule-specific cell types and their progenitors [140]. The crucial role of cytokinin in nodule development and nitrogen fixation was highlighted, with experimental validation of the cytokinin receptor *GmCYTOKININ RESPONSE 1* as essential for these processes [140]. The study revealed the cell-type-specific expression of genes involved in ureides synthesis, emphasizing the compartmentalization of biochemical reactions within nodules [140]. RNA velocity analysis provided insights into the dynamic differentiation trajectories of nodule cells, identifying driver genes such as *GmRESPIRATORY BURST OXIDASE HOMOLOG A*, *GmNODULE INCEPTION 1*, *GmBASIC HELIX-LOOP-HELIX93*, and *GmSCARECROW-LIKE PROTEIN 1* [140]. This high-resolution atlas enhances our understanding of the metabolic and developmental mechanisms underlying soybean nodulation and nitrogen fixation, offering potential strategies for agronomic improvement of legume crops [140]. By manipulating these key genes, it may be possible to develop soybean varieties with improved nitrogen fixation capabilities, reducing the need for synthetic fertilizers and enhancing crop sustainability [140].

The application of scRNA-seq to starch-rich tissues like cassava tuberous roots has provided valuable insights into their development and genetic improvement [141]. Researchers successfully prepared single-cell suspensions by removing free starch particles and obtained high-quality transcriptome data [141]. Various cell clusters were identified, including rare cell types like the Casparian strip, and their differentiation trajectories were mapped using pseudotime analysis [141]. The study revealed that the Casparian strip differentiates from the endodermis and shares lignification-related genes with the xylem, highlighting their similar cell wall components [141]. This single-cell atlas provides valuable insights into the development and genetic improvement of cassava tuberous roots, demonstrating the feasibility of applying scRNA-seq to starch-rich plant tissues and offering new targets for crop enhancement [141]. By targeting these specific cell types and regulatory pathways, breeders can develop cassava varieties with improved root structure and starch content, enhancing both yield and nutritional value [141].

**Figure 2:**
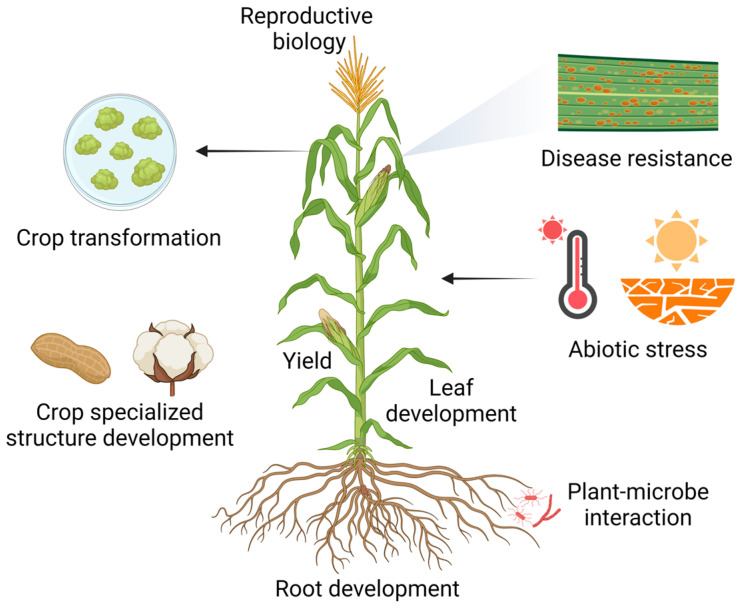
Areas of crop improvement utilizing single-cell and spatial transcriptomics technologies. Single-cell and spatial transcriptomics technologies have been applied to various processes related to crop improvement, including studying the somatic embryogenesis process used for crop transformation, discovering novel resistance or susceptibility genes for plant diseases, gaining insights into improving plant responses to abiotic stresses such as high temperature and drought, investigating the development of yield trait-related organs such as seeds and inflorescence meristems, understanding the interaction between the plant and beneficial microbes in the soil, examining the development of crop specialized structures directly related to yields, such as peanut pegs and cotton fiber cells, and studying general plant development processes, including the development of leaves, roots, and reproductive organs. Created in BioRender. Deschamps, S. (2024) BioRender.com/h01n414 (accessed on 9 October 2024).

The advent of single-cell and spatial transcriptomics technologies has enabled high-resolution gene expression analysis at the single-cell level, along with information on the native spatial localization of cells [142]. Different cell types within plant tissues exhibit distinct responses, necessitating the use of advanced technologies for a more detailed understanding of plant development and environmental interactions. These technologies offer unique opportunities for crop improvement across various areas [47]. In crop transformation, the process of somatic embryogenesis is heterogeneous, involving both embryogenic and non-embryogenic callus formation [143]. Single-cell transcriptomics has been used for comparing the gene expression patterns between these two callus types and identifying genes crucial for embryogenic callus formation [67]. These genes could enhance crop transformation, which is vital for generating transgenic traits and delivering gene-editing traits for crop improvement [68]. For disease resistance, pathogen infection often results in significantly variable responses within the infected tissue, with some cells in direct contact with the pathogen and others not [4]. Single-cell and spatial transcriptomics technologies enable the study of immune responses in cells directly interacting with the pathogen, providing localized characterization of key immune processes and identifying new regulators of plant resistance [3]. Regarding abiotic stresses, responses vary between cell types [96]. Single-cell and spatial transcriptomics technologies have revealed cell type-specific responses to stresses such as high temperature and salt, uncovering novel genes important for plant stress responses [49,98]. Yield-related traits focus on tissues like the female reproductive organ and the seed, which are crucial for yield improvement in major crops like maize, soybean, wheat, and rice [100,101]. Both the ear primordium and the kernel in maize contain diverse cell types, highlighting the importance of single-cell resolution and spatial context in uncovering gene expression patterns and identifying yield-related genes [100,101]. Numerous studies using single-cell and spatial transcriptomics have generated cell atlases and characterized general plant development [144]. These studies can identify cell-specific gene expression elements for driving transgene expression in specific cell types, potentially reducing pleiotropic effects associated with constitutive promoters [145]. Additionally, studying the development of crop-specialized structures, such as cotton fiber and peanut peg, can directly impact yield [146,147]. Single-cell and spatial transcriptomics have been applied to these specialized structures, uncovering novel genes that regulate their development [53,54]. Overall, single-cell and spatial transcriptomics are rapidly advancing fields that have been successfully adapted to various crop species, revealing many genes with potential applications in crop improvement (Table 1, Figure 2). The precision and high resolution of these methods hold great promise for the future of crop enhancement.

## 4. Future Perspectives

Crop improvement has traditionally been accomplished through conventional breeding methods to alter plant development and physiology. With the advent of biotechnology, innovative strategies are required to create varieties that are resistant to biotic and abiotic stresses. Tremendous improvements in plant transformation technologies using morphogenic regulators and progress in tissue culture techniques for regeneration have facilitated the accelerated deployment of resistant crops. Understanding the molecular events that play a role in these processes is going to be the key to improving crop performance. Here, we have provided an overview of the application of different single-cell and spatial transcriptomics technologies for crop improvement. The preparation of high-quality single-cell or single-nuclei samples is critical for making this technology successful. Existing technologies for the capture of low-abundance cells are inconsistent at best and may require increased sequencing depth that would result in increased expense as well as analysis demand. However, sequencing technologies are rapidly and continuously improving while becoming more affordable. With an increasing number of single-cell and spatial transcriptomic datasets becoming available for major crops, we can expect to improve the list of cell-type marker gene candidates that will be able to delineate clusters more accurately. Submicron resolution of spatial transcriptomics methodology is currently limited to targeted platforms, and technology development to increase resolution in untargeted, transcriptome-wide platforms would greatly advance crop improvement efforts.

The higher-resolution analyses of genetic data for specific crop tissue types have provided insights into the development of these organs, such as maize ears, at a cellular level. The knowledge gained has the potential to improve yields. Improvement in spatial transcriptomics technologies will enrich our understanding of plant–microbe symbioses, particularly legume–rhizobial symbiosis, by allowing the simultaneous untargeted capture of both prokaryotic and eukaryotic transcripts within the same cells [164].

The fast-paced advances in technologies for spatial transcriptomics give rise to certain challenges associated with the rapid gathering of high-volume data. Sorting through the vast data generated by these techniques to obtain the appropriate answers to specific questions is something that needs further discussion. For example, the heterogeneity of plant molecular responses to different pathogens makes the interpretation of spatial and single-cell transcriptomics data very subjective and will require a careful dissection of specific facets of disease resistance to put the data into perspective. Similarly, abiotic stresses in nature rarely happen in isolation from other stresses, and we will need to develop methods to deconvolute the massive data generated by spatial and single-cell transcriptomics of crop systems being subjected to such stresses. The same challenges hold true for quantitative traits such as crop yield. At some point in the future, efficient integration of crop spatial transcriptome data with other omics data such as proteome, metabolome, or microbiome will better reveal interactions between different biological molecules. These improved, new tools will further reveal the gene regulatory networks associated with phenotypes and complex traits and allow us to manipulate the underlying mechanisms for crop improvement.

## Figures and Tables

**Table 1:** Selected publications uncovering potential genes for crop improvement discovered through single-cell and spatial transcriptomics.

Category	Crop	Year Published	Tissue	Technology	Genes of Interest	Ref.
Crop transformation	Cotton	2022	Hypocotyl-derived somatic embryo	10x Genomics Chromium single-cell RNA-seq; 10x Genomics Visium	*AATP1*, *DOX2*	[148]
Crop transformation	Cotton	2023	Hypocotyl-derived callus	10x Genomics Chromium single-cell RNA-seq	*LAX2*, *LAX1*, *LOX3*	[67]
Crop transformation	Cotton	2024	Hypocotyl-derived callus	10x Genomics Chromium single-cell RNA-seq	*SELTP*	[62]
Disease resistance	Maize	2023	Root	10x Genomics Chromium single-cell RNA-seq	*ZmWOX5b*, *ZmPIN1a*, *ZmPAL6*, *ZmCCoAOMT2*, and *ZmCOMT*	[88]
Disease resistance	Rubber tree	2023	Leaf	10x Genomics Chromium single-cell RNA-seq	*HbCNL2*	[149]
Disease resistance	Tomato	2024	Leaf	10x Genomics Chromium single-cell RNA-seq	*SlSKIP2*	[89]
Abiotic stress	Cotton	2024	Anther	10x Genomics Chromium single nucleus RNA-seq	*QRT3*, *CYP703A2*	[49]
Abiotic stress	Cotton	2024	Root	10x Genomics Chromium single-cell RNA-seq	*GaGH3.6*	[98]
Abiotic stress	Pea	2023	Shoot apex	10x Genomics Chromium single-cell RNA-seq	*FAS1*, *SWI3A*, *SWI3C*	[97]
Abiotic stress	Wheat	2024	Grain	10x Genomics Visium	*TaSUT1*, *TaSuSY2*, *PGI*, *APGL1*, *PGM*	[150]
Yield	Barley	2023	Germinating grain	10x Genomics Visium	*HOR VU3Hr1G080640*, *HOR VU3Hr1G114270*, *HORVU7Hr1G038700*, *HORVU7Hr1G110470*	[151]
Yield	Maize	2021	Ear primordium	10x Genomics Chromium single-cell RNA-seq	*ZmYABBY9*, *ZmTMO5*	[25]
Yield	Maize	2023	Kernel	10x Genomics Visium	*ZmSUT1*, *ZmSUT7*	[50]
Yield	Maize	2024	Ear primordium	Singleron GEXSCOPE single-cell RNA-seq; Stereo-seq	*ZmMADS8*, *ZmMADS14*	[109]
Yield	Maize	2024	Ear primordium	10x Genomics Chromium single nucleus ATAC-seq	*ZmARF4*, *ZmARF18*, *ZmARF20*, *ZmARF30*, *ZmARF10*, *ZmARF23*, *ZmARF25*, *ZmARF36*	[152]
Yield	Maize	2024	Ear and tassel floret	BD Life Sciences Rhapsody single-cell RNA-seq	*SILKLESS 1*	[153]
Yield	Maize	2024	Ear primordium	10x Genomics Chromium single-cell RNA-seq	*ZmPFKB1*, *ZmPFKB2*, *ZmPFKB3*, *ZmADK1*, *ZmADK2*	[154]
Yield	Maize	2024	Endosperm	10x Genomics Chromium single-cell RNA-seq	*EREB108*, *MYBR19*, *MYBR29*	[113]
Yield	Rice	2022	Young inflorescence	BD Life Sciences Rhapsody single-cell RNA-seq	*DWARF TILLER1*, *OsAUX1*	[155]
Yield	Wheat	2024	Grain	BMKGENE BMKMANU S1000	*TaABI3-3B*	[156]
Yield	Wheat	2024	Inflorescence meristem	Resolve BioSciences Molecular Cartography	*VRN1*, *FUL2*, *LFY*	[157]
General development	Cabbage	2022	Leaves	10x Genomics Chromium single-cell RNA-seq	*BrLHCB4.3*, *BrLHCA6*, *BrFIL.1*, *BrYAB3*, *BrYAB5*, *RPEGs*	[123]
General development	Cassava	2024	Leaves	10x Genomics Chromium single-cell RNA-seq	*ABP19A*, *ALDH3F1*, *NADP-MDH*, *CA*, *RBCS*, *DIT1*, *NADP-ME*	[122]
General development	Cotton	2024	Leaves	10x Genomics Chromium single-cell RNA-seq; 10x Genomics Visium	*GHCU*, *KNGH1*, *KNAT1*, *YUC*	[121]
General development	Garlic	2023	Basal plates of the inflorescence	10x Genomics Chromium single-cell RNA-seq	*BBX*, *VIN3*, *GA2ox*	[158]
General development	Maize	2019	Meiocytes	CEL-seq2 single-cell RNA-seq	Meiotic cell cycle genes, ribosomal genes, membrane-associated genes	[132]
General development	Maize	2020	Shoot apical meristem	CEL-seq2 single-cell RNA-seq	*KN1*, DNA methylation genes, DNA repair-related genes	[159]
General development	Maize	2021	Meiocytes and microspores	Single-cell RNA-seq	*RF4*, *LACS1*, *RPK1*, *APX2*	[131]
General development	Maize	2021	Roots	10x Genomics Chromium single-cell RNA-seq	*SHR*, *SCR*	[128]
General development	Maize	2022	Shoot apical meristem	Cartana in situ sequencing	*PLA1*, *CUC2*, *D11-LIKE*	[130]
General development	Maize	2022	leaf bases	10x Genomics Chromium single nucleus RNA-seq	*OST1*, *MYB60*, *MYB96*, *MPK18*, *SLAC1*, *ZmNRT1.2*, *ZmCYP707A4*	[118]
General development	Maize	2022	Mesophyll cells in leaves	10x Genomics Chromium single-cell RNA-seq	*WRKY*, *ERF*, *NAC*, *MYB*, *HSF*, *DOF1*, *PEPC*, *SCR*, *SHR*	[160]
General development	Maize	2023	Leaves and ligules	10x Genomics Chromium single-cell RNA-seq	*WOX3*	[161]
General development	Maize	2024	Ligular region	BMKGENE BMKMANU DG1000 single nucleus RNA-seq	*bHLH30*, *bHLH155*, *LG1*, *LG2*, *ACS7*, *BRD1*	[120]
General development	Maize	2024	Leaf primordia	Resolve Biosciences Molecular Cartography	*WIP6* (*TOO MANY LATERALS*, *TML*), *ZmbHLH105*, *PIN1*	[162]
General development	Maize	2024	Leaves	Resolve Biosciences Molecular Cartography	*PIN1a*, *TML1*, *HB52*, *ARF3a*, *RLD1*	[119]
General development	Peanut	2021	Leaf blade	10x Genomics Chromium single-cell RNA-seq	*RBCS*, *LHCB*, *GSTF*, *ERF*, *NAC*, *AHL23*	[115]
General development	Peanut	2023	Leaves	10x Genomics Chromium single-cell RNA-seq	*FAD2*, *WRKY6*, *ERF109*, *WRKY23*, *ERF6*, *MYB102*, *WRKY30*, *FAD7*, *LOX*, *OPR3*, *CYP735A*, *LOG1*	[116]
General development	Peanut	2024	Leaves	10x Genomics Chromium single nucleus RNA-seq and single nucleus ATAC-seq	*AP2/ERFs*, *KAN4*, *AhAHL11*, *CDF5*, *RGA*	[114]
General development	Peanut	2024	Seedling leaves	10x Genomics Chromium single-cell RNA-seq	*AHL17*, *OsIAA3*, *OsGH3-2*, *PYL2, OsSAPK9*, *OsSAPK10*	[117]
General development	Rice	2017	Mesophyll cells in leaves	Single-cell RNA-seq	*Os09g19700*, *Os01g22900*, *ATML1*	[124]
General development	Rice	2021	Roots	10x Genomics Chromium single-cell RNA-seq	*OsGATA6*, *Os04g0125700*, *Os01g0248900*, Cell differentiation-related genes, chromatin accessibility-related genes	[125]
General development	Rice	2021	Roots	10x Genomics Chromium single-cell RNA-seq	*LOC_Os06g38960*, *LOC_Os03g25280, LOC_Os04g46810*, *LOC_Os01g73700*, *LOC_Os08g03450*, *LOC_Os03g37490*, *LOC_Os01g73980*	[126]
General development	Rice	2023	Pistils	10x Genomics Chromium single nucleus RNA-seq	Calcium signaling genes, RNA processing genes, ATP generation genes	[129]
General development	Rice	2024	Plumules	10x Genomics Chromium single-cell RNA-seq	*BURP*, *LAZY1*, *TAC1*, *OsPS1-F*, *OsPIP1;2*, *FON1*	[133]
General development	Tomato	2022	Shoot-borne and lateral roots	Molecular crowding single-cell RNA barcoding and sequencing	*LBD TFs* (*SBRL*, *RTCS*, *CRL1*), *WOX11*, *PLTs*, auxin and cytokinin response genes	[127]
General development	Wheat	2023	Roots	10x Genomics Chromium single nucleus RNA-seq and single nucleus ATAC-seq	*MYB3R4*, *REF6*, *HDG1*, and *GATAs*, *TaSPL14*	[163]
Specialized structures	Cassava	2023	Tuberous roots	10x Genomics Chromium single-cell RNA-seq	Genes related to Casparian strip, endodermis, xylem, lignification	[141]
Specialized structures	Cotton	2022	Ovules	10x Genomics Chromium single-cell RNA-seq	*HD1*, *PDF2*, *MYB25-like*, *HOX3*	[54]
Specialized structures	Cotton	2023	Leaves	10x Genomics Chromium single-cell RNA-seq	*GoHSFA4a*, *GoNAC42*, *GoPGF*, *GhDIR5*, *GhDIR6*	[134]
Specialized structures	Cotton	2023	Leaves	10x Genomics Chromium single-cell RNA-seq	*GoPGF*, *AP2/ERFs*, *bHLHs*, *C2H2s*, *GRASs*, *MYBs*, *NACs*, *WRKYs*, *GbiERF114*, *GbiZAT11*, *GbiNTL9*	[135]
Specialized structures	Cotton	2023	Cotyledons	10x Genomics Chromium single-cell RNA-seq	*GoPGF*, *GhERF105*, *ERF13*, *MYB14*, *GH_A05G3906*, *GhJUB1*	[136]
Specialized structures	Cotton	2023	Ovules	10x Genomics Chromium single-cell RNA-seq and single nucleus ATAC-seq	*GhRALF1*, *GhTCP14*, *PRRs*, *CCA1*	[137]
Specialized structures	Peanut	2022	Roots, stems, hypocotyls, pegs	Stereo-seq	*BBI1*, *MADS1*, *AGL5*	[138]
Specialized structures	Peanut	2024	Pegs, pods	10x Genomics Chromium single nucleus RNA-seq and single nucleus ATAC-seq	*PINs*, *NPY5s*, *SGR6s*, *IDD14*, *ABCI11*, *ABCB15*, *GTE1*, *PHOT1*, *SPA1*, *YAB5*, *IAA9*, *AGL1*, *AGL5*	[53]
Specialized structures	Soybean	2022	Root Nodules	10x Genomics Chromium single nucleus RNA-seq; Stereo-seq	Ureide biogenesis genes, *GLYMA_02G004800*	[139]
Specialized structures	Soybean	2023	Root Nodules	10x Genomics Chromium single nucleus RNA-seq	*GmCRE1*, *GmRbohA*, *GmNIN1a*, *GmbHLH93*, *GmSCL1*	[140]

## Data Availability

No new data were created or analyzed in this study. Data sharing is not applicable to this article.
